# Associations between locus coeruleus integrity and nocturnal awakenings in the context of Alzheimer’s disease plasma biomarkers: a 7T MRI study

**DOI:** 10.1186/s13195-021-00902-8

**Published:** 2021-09-24

**Authors:** Maxime Van Egroo, Roy W. E. van Hooren, Heidi I. L. Jacobs

**Affiliations:** 1 Faculty of Health, Medicine and Life Sciences, School for Mental Health and Neuroscience, Alzheimer Centre Limburg, Maastricht University, UNS40 box 34, P.O. Box 616, 6200 MD Maastricht, The Netherlands; 2 Faculty of Psychology and Neuroscience, Department of Cognitive Neuroscience, Maastricht University, Maastricht, The Netherlands; 3 Gordon Center for Medical Imaging, Department of Radiology, Massachusetts General Hospital and Harvard Medical School, Boston, MA USA

**Keywords:** Alzheimer’s disease, Locus coeruleus, Ultra-high field neuroimaging, Subjective sleep metrics, Blood-based Alzheimer’s disease biomarkers

## Abstract

**Background:**

The brainstem locus coeruleus (LC) constitutes the intersection of the initial pathophysiological processes of Alzheimer’s disease (AD) and sleep-wake dysregulation in the preclinical stages of the disease. However, the interplay between in vivo assessment of LC degeneration and AD-related sleep alterations remains unknown. Here, we sought to investigate whether MRI-assessed LC structural integrity relates to subjective sleep-wake measures in the context of AD plasma biomarkers, in cognitively unimpaired older individuals.

**Methods:**

Seventy-two cognitively unimpaired older individuals aged 50–85 years (mean age = 65.2 ± 8.2 years, 37 women, 21 *APOE* ε4 carriers) underwent high-resolution imaging of the LC at 7 Tesla, and LC structural integrity was quantified using a data-driven approach. Reports on habitual sleep quality and nocturnal awakenings were collected using sleep questionnaires. Plasma levels of total tau, p-tau_181_, Aβ_40_, and Aβ_42_ were measured using single-molecule array technology.

**Results:**

Intensity-based cluster analyses indicated two distinct LC segments, with one covering the middle-to-caudal LC and displaying lower intensity compared to the middle-to-rostral cluster (*t*_70_ = −5.12, *p* < 0.0001). After correction for age, sex, depression, and *APOE* status, lower MRI signal intensity within the middle-to-caudal LC was associated with a higher number of self-reported nocturnal awakenings (*F*_1,63_ = 6.73, *p*_FDR_ = 0.03). Furthermore, this association was mostly evident in individuals with elevated levels of total tau in the plasma (*F*_1,61_ = 4.26, *p* = 0.04).

**Conclusion:**

Our findings provide in vivo evidence that worse LC structural integrity is associated with more frequent nocturnal awakenings in the context of neurodegeneration, in cognitively unimpaired older individuals. These results support the critical role of the LC for sleep-wake regulation in the preclinical stages of AD and hold promises for the identification of at-risk populations for preventive interventions.

**Supplementary Information:**

The online version contains supplementary material available at 10.1186/s13195-021-00902-8.

## Background

In the worldwide effort to identify leverage points to delay the onset of Alzheimer’s disease (AD), sleep has emerged as a potent modifiable factor to slow down the characteristic pathophysiological processes of the disease, i.e., the accumulation of amyloid-beta (Aβ) and tau proteins, together with neurodegeneration [1]. Two recent meta-analyses of, respectively, 27 observational and 18 longitudinal studies reported that individuals with sleep-related issues are at a ~1.5 times increased risk of developing AD and that an estimated 15% of AD in the population may be linked to treatable sleep problems [2, 3].

One of the critical brain regions involved in both initial AD pathogenesis and sleep-wake regulation is a small nucleus located in the brainstem: the locus coeruleus (LC). Landmark post-mortem studies have demonstrated that the LC is among the first sites of tau pathology [4], starting as early as age thirty [5]. As part of the complex ascending arousal system, the LC regulates wakefulness periods, arousal, and various cognitive processes, by supplying norepinephrine to the entire cortex [6]. During a typical sleep cycle, LC neurons become progressively silent, which ensures overall consolidation of the sleep period and normal transitions across sleep stages [7], while its activity underlies sleep-to-wake transitions [8, 9].

In animal studies, chronic sleep disruption of 3 days a week during 1 month was enough to induce long-lasting alterations in LC neurons morphology (i.e., reductions in neuronal count and axonal projections) [10, 11] and to promote tau accumulation in the LC [12]. In humans, significant degeneration of wake-promoting LC neurons concomitant with increased tau protein burden was reported at histological investigation of AD brains, which was suggested to contribute to the disrupted sleep-wake pattern commonly experienced by those patients [13, 14]. However, no direct assessments of sleep-wake measures were available in these post-mortem studies, leaving important questions on the interplay between LC alterations, sleep-wake patterns, and AD-related pathophysiological processes unanswered. As both sleep-wake disruption and LC pathologic processes can be detected as early as in the 5th or 6th decade of life [15, 16], addressing these gaps in cognitively unimpaired older individuals may provide new insights into the pathological processes in the earliest stages of AD.

To our knowledge, no studies have directly related in vivo LC measures to sleep-wake variables, most likely because it is challenging to image this brain nucleus, due to its deep location in the brainstem and its small size (~15 mm long and 2 × 2 mm thick). However, the development of novel MRI methods has enabled in-depth assessment of its properties in vivo [17]. Here, we used state-of-the-art methods in ultra-high field neuroimaging to investigate LC structural integrity in vivo in a cohort of cognitively unimpaired older individuals and to relate it to participants’ subjective evaluations of sleep quality and nocturnal awakenings. In addition, we sought to examine interactive effects with early AD-related pathophysiological processes, as measured with recently developed blood-based biomarkers, in order to evaluate LC integrity as a potential early marker for individuals with AD-related sleep disturbances.

## Material and methods

### Participants

Seventy-two cognitively unimpaired older individuals aged 50–85 years (mean age = 65.2 ± 8.2 years, 37 women) were recruited to participate in this study (Table 1). The main exclusion criteria were contraindications for ultra-high field neuroimaging, performance on key cognitive tests 2 standard deviations below the mean (according to normative data corrected for age, sex, and education), past or present psychiatric or neurological disorders, major vascular disorders, left-handedness, use of drugs or psychoactive medication, and excessive alcohol consumption (> 15 units/week).

**Table 1:** Sample characteristics (mean ± SD)

	*N* = 72
Age (years)	65.2 ± 8.2
Sex (*N*)	37F/35M
Ethnicity	Caucasian
Right-handed (*N*, %)	72 (100)
Body mass index (kg/m^2^)	25.5 ± 4.0
MMSE (score)	28.9 ± 1.2
*APOE* ε4 carriers (*N*, %)	21 (29)
Total tau (pg/ml)	2.5 ± 0.7
P-tau_181_ (pg/ml)	1.7 ± 0.5
Aβ_40_ (pg/ml)	228.9 ± 33.6
Aβ_42_ (pg/ml)	12.1 ± 2.3
Subjective sleep quality (total GSQS score)	3.3 ± 3.6
Nocturnal awakenings (self-reports)	1.8 ± 1.2

### 7T MRI acquisition and pre-processing

All participants underwent high-resolution imaging of the brainstem on a 7T Magnetom Siemens scanner (Siemens Healthineers, Erlangen, Germany) using a magnetization transfer-weighted turbo flash (MT-TFL) sequence particularly sensitive to LC-related contrast [18, 19], with a field-of-view placed perpendicular to the pons and covering the area between the inferior colliculus and the inferior border of the pons. Importantly, the LC-related MRI signal obtained with this sequence has been established to reflect LC neuronal and fiber projection density [20].

Our 7T LC MRI pre-processing pipeline is summarized in Supplementary Figure 1, and details are available in the Supplementary Methods. In brief, intensity-normalized images were obtained by dividing individual MT-TFL images by the subject-specific mean intensity of a 10 × 10 voxel region-of-interest located in the pontine tegmentum (PT). Next, a study-specific template was built based on all individual intensity-normalized MT-TFL images. The LC was manually delineated on the resulting template in the common space, based on voxel intensities and the known LC anatomy. This LC mask was then applied on each intensity- and spatially normalized individual image on a per hemisphere basis as previously described [18, 21], to extract MRI signal values for subsequent cluster analyses. As in previous work [22], intensity in the PT region served as control in post hoc analyses.

### LC MRI signal cluster analysis

Cluster analyses were performed to identify sub-portions within the LC structure in a data-driven manner. We first determined the optimal number of clusters using ‘*evalcluster*’ function implemented in MATLAB2017b (The Mathworks Inc., Natick, MA, USA) based on the Calinski-Harabasz criterion. The optimal solution was then used as a prior to run a K-means algorithm (‘*kmeans*’ function in MATLAB, Euclidean distance, 100 iterations) on median MRI signal intensity within each slice of the LC mask, separately for left and right hemisphere. Median MRI signal intensity values in each identified cluster constituted our primary measures of interest and were computed for left and right LC and also averaged over both hemispheres. Consistent with previous work [16, 21], we also computed additional LC metrics to conduct post hoc sensitivity analyses, including the mean and peak intensity in each cluster, and mean MRI signal intensity in three manually defined equidistant sections of the LC.

### Subjective sleep quality assessment

Sleep quality was investigated through participants’ self-reports on the quality of their sleep during a representative night, using the Groningen Sleep Quality Scale (GSQS) [23]. The GSQS is a sleep quality questionnaire widely used in the Netherlands, which comprises 15 dichotomous (yes/no) items related to different dimensions of sleep throughout a habitual night (i.e., sleep latency, sleep duration, feeling of restlessness, etc.). The GSQS scores strongly correlate with the Pittsburgh Sleep Quality Index, another subjective sleep quality scale traditionally used by sleep researchers (unpublished data, *n* = 61 cognitively unimpaired older individuals, *r* = 0.79). Subjective sleep quality was computed as the sum of the scores to each GSQS item, with a higher score reflecting an overall worse sleep quality. An additional item was used to measure the habitual number of self-reported nocturnal awakenings (‘I wake up on average _ times during the night’).

### Alzheimer’s disease blood-based biomarker assessment

EDTA plasma samples were obtained through venipuncture (fasted). Samples were centrifuged at 2000 × g, aliquoted in polypropylene tubes, and stored at −80°C in our biobank within 60 min of collection. Plasma was analyzed using ultra-sensitive single-molecule array technology of the automated Simoa HD-1 analyzer with the Simoa Human Neurology 3-Plex A assay kit (Quanterix, Lexington, KY, USA) that simultaneously measures plasma concentrations of Aβ_40_, Aβ_42_, and total tau. Analyses were performed in duplicates (mean % coefficient of variation [%CV]: Aβ_40_ 3.8%CV, Aβ_42_ 4.1%CV, total tau 6.7%CV) using a 1:4 automated dilution protocol. The levels of tau phosphorylated at threonine 181 (p-tau_181_) were measured using the Simoa Human tau immunoassay kits on the Simoa HD-1 Analyzer (7.1%CV). *APOE* genotyping was further performed using polymerase chain reaction based on blood sample DNA extraction. Participants’ *APOE* status was defined as ‘ε4 carrier’ if they carry at least one ε4 allele.

### Statistical analysis

Statistical analyses were conducted in SAS 9.4 (SAS Institute, NC, USA) using generalized linear mixed model (GLMM). Prior to model fitting, the distribution of the dependent variable in each GLMM was determined in MATLAB2017b, and models were adjusted accordingly. All statistical models included age, sex, and *APOE* status as covariates. Models with sleep metrics as the dependent variable were further adjusted for depression scores, as measured by the Hamilton Depression Rating Scale [24]. Subject (intercept) was set as a random factor. *p*-values derived from these main analyses linking sleep-wake metrics to LC intensity were corrected for multiple comparisons using the false discovery rate (FDR) approach. LC intensity variables showing significant associations with sleep metrics were further tested in models including the interaction term with blood-based AD biomarkers. Post hoc control analyses were conducted using subject-specific median intensity in a PT region-of-interest. Post hoc sensitivity analyses were performed using additional LC metrics of peak cluster intensity, mean cluster values, and mean values in three equidistant LC sections. Degrees of freedom were estimated using Kenward-Roger’s correction. In all GLMMs, effect sizes for significant effects were estimated with semi-partial *R*^*2*^ (*R*^2^_β*_) values [25].

## Results

### Association between demographics, subjective sleep measures, and blood-based biomarkers

After adjusting for age, women reported worse sleep quality (*F*_1,69_ = 5.54, *p* = 0.02, *R*^2^_β*_ = 0.07) and more nocturnal awakenings (*F*_1,69_ = 4.75, *p* = 0.03, *R*^2^_β*_ = 0.06) compared to men. In addition, higher depression scores, although in the subclinical range for all individuals, were associated with worse sleep quality (*F*_1,68_ = 27.22, *p* < 0.0001, *R*^2^_β*_ = 0.29) and higher number of nocturnal awakenings (*F*_1,68_ = 7.91, *p* = 0.006, *R*^2^_β*_ = 0.10), after adjusting for age and sex. With regard to plasma measures, the range of values was consistent with previous Simoa-based studies in cognitively unimpaired older individuals [26, 27]. Older age was associated with higher plasma levels of p-tau_181_ (*F*_1,66_ = 8.36, *p* = 0.005, *R*^2^_β*_ = 0.11) and Aβ_40_ (*F*_1,66_ = 12.35, *p* < 0.001, *R*^2^_β*_ = 0.16). *APOE* ε4 carriers (29%) displayed lower levels of Aβ_42_ (*F*_1,66_ = 10.48, *p* = 0.002, *R*^2^_β*_ = 0.14) and at-trend level higher plasma levels of p-tau_181_ (*F*_1,66_ = 3.44, *p* = 0.07) compared to non-carriers (Supplementary Figure 2). No significant relationships were observed between plasma biomarkers and subjective measures of sleep quality or nocturnal awakenings (Supplementary Figure 3 and Supplementary Table 1).

### Identification of clusters in LC MRI signal intensity

**Fig. 1:**
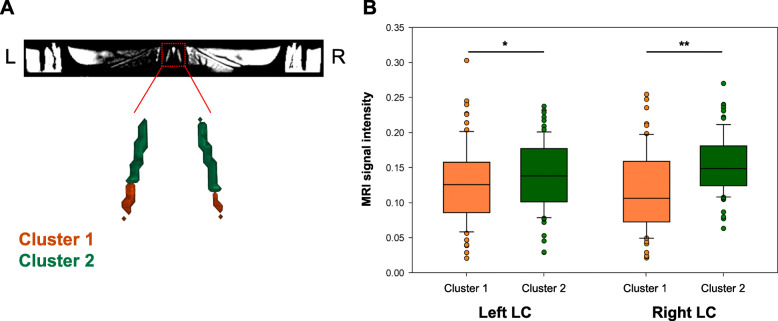
Intensity-based cluster analyses of the LC structure. **A** Cluster analyses on MRI signal intensity within the LC mask revealed two distinct clusters along the LC structure, one covering the middle-to-caudal LC (orange, cluster 1) and the other covering the middle-to-rostral LC (green, cluster 2). **B** Box plots of MRI signal intensity within clusters 1 and 2, represented by hemisphere. Paired *t* test analyses showed that MRI signal intensity in cluster 1 is significantly lower than that in cluster 2. *p < .05, **p < .001

Cluster analyses on LC MRI signal intensity revealed two clusters, one covering the middle-to-caudal portion (bottom 7 slices for left LC and bottom 5 slices for right LC) and the other cluster covering the middle-to-rostral part (top 12 slices for left LC, top 14 slices for right LC, Fig. 1A). Paired *t* test analyses showed that median intensity within the middle-to-caudal cluster was significantly lower than in the middle-to-rostral cluster when considered bilaterally (*t*(70) = −5.12, *p* < 0.0001), but also when tested separately for both hemispheres (left: *t*(70) = −2.23, *p* = 0.03; right: *t*(68) = −6.46, *p* < 0.0001, Fig. 1B).

### Association between LC intensity and subjective sleep measures

**Fig. 2:**
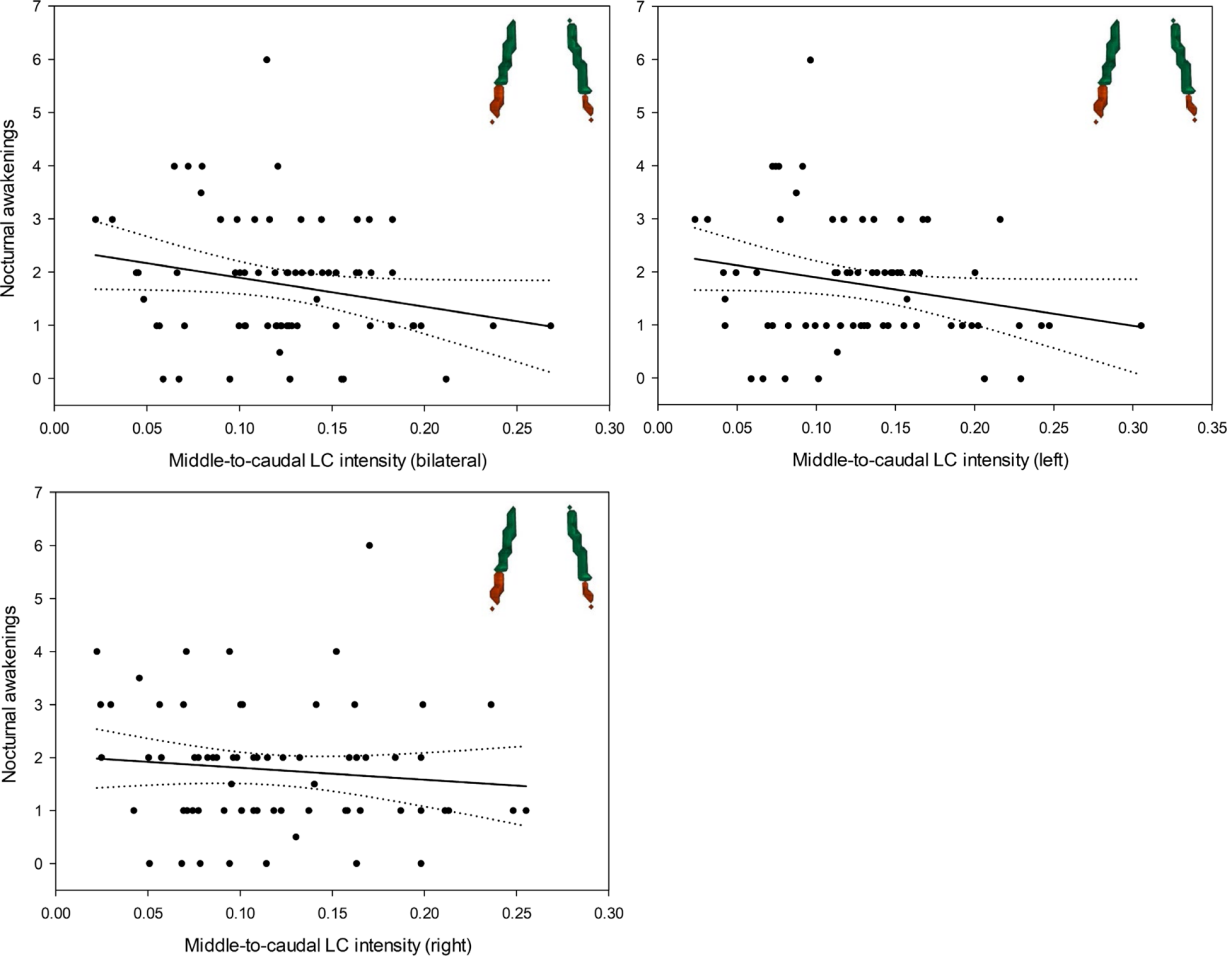
Associations between subjective reports of nocturnal awakenings and middle-to-caudal LC structural integrity, considered bilaterally (top left), in the left hemisphere (top right), and in the right hemisphere (bottom left). Simple regression lines are used for a visual display and do not substitute the GLMM outputs. Dotted lines represent 95% confidence intervals of these simple regressions

After adjusting for age, sex, *APOE* status, and depression scores, subjective sleep quality was not associated with MRI signal intensity values for either of the two LC clusters (Supplementary Table 2). However, we found a significant negative relationship between intensity within the middle-to-caudal LC cluster and participants’ reports of nocturnal awakenings (*F*_1,63_ = 5.85, *p*_FDR_ = 0.03, *R*^2^_β*_ = 0.09, Fig. 2, Table 2), which was strongest in the left hemisphere (*F*_1,63_ = 6.73, *p*_FDR_ = 0.03, *R*^2^_β*_ = 0.10). These associations were not observed when investigating the middle-to-rostral cluster (Table 2).

**Table 2:** GLMM outputs of the associations between subjective reports of nocturnal awakenings and middle-to-caudal (top) or middle-to-rostral (bottom) LC structural integrity, considered bilaterally (model 1), for left LC (model 2), and for right LC (model 3)

	***Model 1***	***Model 2***	***Model 3***
Middle-to-caudal LC integrity	*F*_1,63_ = 5.85 *p*_FDR_ = 0.03 *R*^*2*^_β*_ = 0.09	*F*_1,63_ = 6.73 *p*_FDR_ = 0.03 *R*^*2*^_β*_ = 0.10	*F*_1,63_ = 0.71 *p*_FDR_ = 0.40
Age	*F*_1,63_ = 3.27 *p* = 0.08	*F*_1,63_ = 4.08 *p* = 0.05	*F*_1,63_ = 2.20 *p* = 0.14
Sex	*F*_1,63_ = 6.67 *p* = 0.01 *R*^*2*^_β*_ = 0.10	*F*_1,63_ = 7.76 *p* = 0.007 *R*^*2*^_β*_ = 0.11	*F*_1,63_ = 6.02 *p* = 0.02 *R*^*2*^_β*_ = 0.09
Depression	*F*_1,63_ = 8.07 *p* = 0.006 *R*^*2*^_β*_ = 0.11	*F*_1,63_ = 7.26 *p* = 0.009 *R*^*2*^_β*_ = 0.10	*F*_1,63_ = 8.42 *p* = 0.005 *R*^*2*^_β*_ = 0.12
*APOE* status	*F*_1,63_ = 2.16 *p* = 0.15	*F*_1,63_ = 2.29 *p* = 0.14	*F*_1,63_ = 2.46 *p* = 0.12
Middle-to-rostral LC integrity	*F*_1,63_ = 1.37 *p*_FDR_ = 0.37	*F*_1,63_ = 2.03 *p*_FDR_ = 0.37	*F*_1,62_ = 0.36 *p*_FDR_ = 0.55
Age	*F*_1,63_ = 2.70 *p* = 0.11	*F*_1,63_ = 2.68 *p* = 0.11	*F*_1,62_ = 2.43 *p* = 0.12
Sex	*F*_1,63_ = 6.09 *p* = 0.02 *R*^*2*^_β*_ = 0.09	*F*_1,63_ = 6.60 *p* = 0.01 *R*^*2*^_β*_ = 0.09	*F*_1,62_ = 5.53 *p* = 0.02 *R*^*2*^_β*_ = 0.08
Depression	*F*_1,63_ = 7.11 *p* = 0.01 *R*^*2*^_β*_ = 0.10	*F*_1,63_ = 7.40 *p* = 0.008 *R*^*2*^_β*_ = 0.11	*F*_1,62_ = 6.86 *p* = 0.01 *R*^*2*^_β*_ = 0.10
*APOE* status	*F*_1,63_ = 2.38 *p* = 0.13	*F*_1,63_ = 2.43 *p* = 0.12	*F*_1,62_ = 1.90 *p* = 0.17

We then performed several sensitivity analyses using different quantifications of LC MRI signal intensity (cluster mean intensity, cluster peak intensity, and mean intensity within three equidistant LC sections), and we found similar associations using mean values within the middle-to-caudal cluster for both bilateral (*F*_1,63_ = 4.43, *p* = 0.04, *R*^2^_β*_ = 0.07) and left LC (*F*_1,63_ = 5.04, *p* = 0.03, *R*^2^_β*_ = 0.07). Peak intensity in the middle-to-caudal cluster also yielded comparable results for bilateral (*F*_1,63_ = 5.17, *p* = 0.03, *R*^2^_β*_ = 0.08) and left LC (*F*_1,63_ = 6.31, *p* = 0.01, *R*^2^_β*_ = 0.09). Finally, delineation of the LC in three equivalent segments further supports the specific associations between the caudal part of the LC and nocturnal awakenings for both the bilateral (*F*_1,62_ = 4.15, *p* = 0.05, *R*^2^_β*_ = 0.06) and left LC (*F*_1,63_ = 5.26, *p* = 0.03, *R*^2^_β*_ = 0.08).

In our control analysis, we found no significant association between nocturnal awakenings and median PT values (*F*_1,63_ = 0.91, *p* = 0.34), supporting specificity in the relationships observed above with LC intensity metrics.

### Interactive effect with blood-based AD biomarkers

**Fig. 3:**
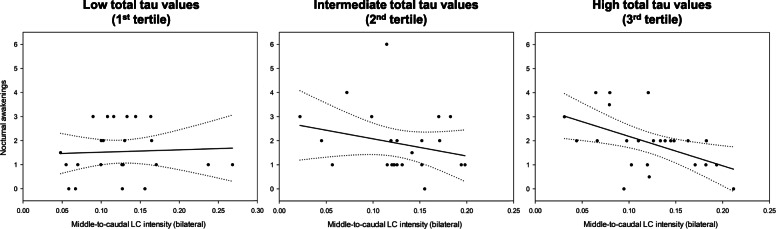
Interactive effect of middle-to-caudal LC structural integrity and plasma total tau levels. The association between subjective reports of nocturnal awakenings and middle-to-caudal LC structural integrity is represented by tertiles of plasma total tau levels. Simple regression lines are used for a visual display and do not substitute the GLMM outputs. Dotted lines represent 95% confidence intervals of these simple regressions

Finally, we investigated whether the relationships between LC intensity and nocturnal awakenings are modified by plasma AD biomarkers, and found that the negative relationship between middle-to-caudal LC intensity and nocturnal awakenings was particularly evident in individuals with higher levels of total tau (*F*_1,61_ = 4.23, *p* = 0.04, *R*^2^_β*_ = 0.06, Fig. 3, Table 3). This interaction was predominantly present in the left hemisphere (*F*_1,61_ = 4.26, *p* = 0.04, *R*^2^_β*_ = 0.07). No significant interaction was observed with any of the other plasma biomarkers (Supplementary Table 3).

**Table 3:** GLMM outputs of the associations between subjective reports of nocturnal awakenings and the interaction term total tau*middle-to-caudal LC structural integrity, considered bilaterally (model 1), for left LC (model 2), and for right LC (model 3)

	***Model 1***	***Model 2***	***Model 3***
Total tau*middle-to-caudal LC integrity	*F*_1,61_ = 4.23 *p* = 0.04 *R*^*2*^_β*_ = 0.06	*F*_1,61_ = 4.26 *p* = 0.04 *R*^*2*^_β*_ = 0.07	*F*_1,59_ = 1.96 *p* = 0.17
Middle-to-caudal LC integrity	*F*_1,61_ = 1.47 *p* = 0.23	*F*_1,61_ = 1.55 *p* = 0.22	*F*_1,59_ = 1.20 *p* = 0.28
Total tau	*F*_1,61_ = 3.12 *p* = 0.08	*F*_1,61_ = 3.17 *p* = 0.08	*F*_1,59_ = 1.05 *p* = 0.31
Age	*F*_1,61_ = 4.08 *p* = 0.05	*F*_1,61_ = 5.01 *p* = 0.03 *R*^*2*^_β*_ = 0.08	*F*_1,59_ = 2.61 *p* = 0.11
Sex	*F*_1,61_ = 6.84 *p* = 0.01 *R*^*2*^_β*_ = 0.10	*F*_1,61_ = 7.90 *p* = 0.007 *R*^*2*^_β*_ = 0.11	*F*_1,59_ = 5.59 *p* = 0.02 *R*^*2*^_β*_ = 0.09
Depression	*F*_1,61_ = 7.94 *p* = 0.007 *R*^*2*^_β*_ = 0.12	*F*_1,61_ = 6.64 *p* = 0.01 *R*^*2*^_β*_ = 0.10	*F*_1,59_ = 9.35 *p* = 0.003 *R*^*2*^_β*_ = 0.14
*APOE* status	*F*_1,61_ = 1.83 *p* = 0.18	*F*_1,61_ = 2.19 *p* = 0.14	*F*_1,59_ = 2.24 *p* = 0.14

## Discussion

Sleep-wake disruption constitutes a hallmark of the aging process [28] and contributes to the unfolding of AD, as early as during the preclinical stages of the disease [29, 30]. Given the particular involvement of the LC in both sleep-wake mechanisms and initial AD pathogenesis, it may constitute a strong candidate as a neurobiological correlate of the sleep-wake dysregulation observed in the earliest stages of the disease. Here, we provide in vivo evidence that worse integrity of the middle-to-caudal LC structure is associated with increased number of self-reported nocturnal awakenings in cognitively unimpaired older individuals and that this relationship may be exacerbated in individuals with elevated levels of total tau in the plasma. These results expand on the important contribution of the LC to sleep-wake regulation and have implications for the early detection of sleep disturbances in older individuals at higher risk for dementia.

Our findings that LC structural integrity specifically relates to nocturnal awakenings, but not to a broader metric of sleep quality, corresponds to the role of the wake-promoting LC neurons as a critical component of the ascending arousal system to regulate arousal and wakefulness periods during sleep [9, 31]. While it may seem counterintuitive that worse LC integrity—possibly reflecting volumetric changes including shrinking of wake-promoting neurons—would be associated with an increased number of awakenings, we speculate that compensatory mechanisms are triggered within intact neurons leading to hyperactivity of the LC among the sleep-wake circuitry. Indeed, increased firing frequency and irregular firing patterns have been observed in the remaining neurons following neurotoxin-induced LC reduction in mice [32]. Accordingly, elevated levels of 3-methoxy-4-hydroxyphenylethyleneglycol (MHPG), the principal metabolite of norepinephrine, have been reported after experimental lesions of the LC in rats [33]. Moreover, several studies in humans have suggested that elevated MHPG constitutes a detrimental process contributing to AD-related pathological changes in the early stages of the disease [34–37]. In that context, we propose that dysregulated norepinephrine release due to aberrant LC activity during sleep, and especially when the LC neurons are supposed to be almost completely quiescent—such as during rapid eye movement sleep [38]—would lead to an imbalance in the interplay between wake- and sleep-promoting neurons [39, 40], resulting in more frequent and inappropriate awakenings.

Through a series of sensitivity analyses, we provided additional evidence that the associations between LC integrity and nocturnal awakenings arise specifically from the cluster-based middle-to-caudal part. Interestingly, a recent study reported similar regional associations between MRI-based assessments of LC structure and subjective measures of daytime dysfunction in 481 older men from the Vietnam Era Twin Study of Aging [22]. It is important to note that the caudal portion of the LC is known to be more difficult to accurately image due to its diffuse anatomical organization [21, 41]. As such, previous work has suggested that caudal sections of the LC on structural MR images likely correspond to the middle part of the actual LC [16]. Consistent with our observation of a stronger negative LC integrity-awakening relationship at elevated total tau levels, autopsy work reported that this middle portion of the LC structure exhibits greater vulnerability to tau accumulation in early Braak stages [42]. Future studies are warranted to replicate this topographical specificity and uncover a potential wakefulness-specific modular architecture in the LC [6]. Nevertheless, our control analysis revealed no significant relationship with MRI signal measured in a PT region-of-interest, located close to the LC. This supports the regional specificity of the LC in the observed associations, thereby reducing the likelihood of having grasped spurious correlations.

Our results suggest that the association between worse LC integrity and higher number of self-reported nocturnal awakenings is more marked in individuals with elevated levels of total tau in the plasma. However, we did not observe similar interactive effects with the more specific blood-based AD biomarkers of p-tau_181_, Aβ_40_, and Aβ_42_. Although plasma total tau values also encompass phosphorylated forms of tau protein, a recent meta-analysis of blood-based biomarkers suggested that total tau is rather a marker of neurodegeneration, with better ability to discriminate between AD patients and controls in studies using Simoa techniques compared to traditional ELISA methods [43]. Even though, based on autopsy studies, we would expect phosphorylated tau accumulation to contribute to the integrity values of our LC assessment, it is possible that the plasma p-tau_181_ in our cohort of healthy individuals may not be sensitive enough to the earliest tau pathological changes. Plasma p-tau_231_ has been recently put forward as a promising biomarker to discriminate individuals in early Braak stages and with sub-threshold Aβ markers [44], which cannot be achieved with p-tau_181_. Taking advantage of these recent, more sensitive plasma tau markers may therefore reveal distinct associations, especially in studies of healthy older individuals. Our findings should thus be interpreted in the broader context of neuronal injury and increased risk for cognitive decline as well as incident dementia [45], including but not limited to AD.

### Limitations

The strengths of our study include state-of-the-art investigation of the LC structure in vivo, using ultra-high field imaging combined with a data-driven approach to examine associations between nocturnal awakenings and fine-grained interindividual variability in LC morphology in cognitively unimpaired older individuals. However, our study also bears limitations. First, the cross-sectional approach restricts causal interpretation, although bidirectional relationships between sleep-wake disruption and LC alteration are likely at play [29]. While longitudinal observations can provide information about the temporal chain of events, interventional designs that interfere with sleep-wake regulation and/or LC activity are needed to address causality. In addition, previous studies reported a female vulnerability to AD pathology and AD-related cognitive decline [46, 47], and a recent review suggested a similar sex-specific vulnerability for the LC-norepinephrine system [48]. While we and others observed more subjective reports of nocturnal awakenings in women compared to men, we did not see sex differences in LC integrity measures, consistent with previous in vivo LC MRI studies in cognitively unimpaired individuals [16, 21, 49]. As the effect of sex may be differentially expressed along the clinical continuum of the disease [50], it will thus be important to further examine the relationships between sex-specific vulnerability to AD, sleep metrics, and LC integrity in populations including cognitively impaired individuals. Finally, future studies should include objective assessments of sleep-wake regulation, such as actigraphic or EEG recordings, alongside subjective measurements, to allow for refined quantification of sleep-wake metrics. This would also contribute to further identifying which characteristics of nocturnal awakenings (e.g., timing, duration) are most closely related to LC structural integrity.

## Conclusions

We provide in vivo evidence that more frequent self-reported nocturnal awakenings are associated with worse structural integrity of the LC in cognitively unimpaired older individuals, particularly in those with elevated plasma markers of neurodegeneration. Thus, the investigation of sleep-wake disruption and LC structural integrity in aging may constitute interesting targets to identify individuals at higher risk of developing dementia and holds promises for preventive interventions mitigating the effect of sleep disturbances on brain physiology.

## Supplementary Information

**Additional file 1.** Supplementary methods, figures, and tables.

## Data Availability

Participants did not explicitly consent to their data being made public, and therefore, access to their demographic, raw, or processed imaging data is restricted. Requests for the anonymized data should be made to Heidi Jacobs (http://www.heidijacobs.nl; h.jacobs@maastrichtuniversity.nl or hjacobs@mgh.harvard.edu) and will be reviewed by an independent data access committee, taking into account the research proposal and intended use of the data. Data domains in which data collection is ongoing can only be shared under these regulations once data collection and quality assessment are completed. Requestors are required to sign a data sharing agreement to ensure participants’ confidentiality is maintained prior to the release of any data and that procedures conform with the EU legislation on the general data protection regulation and local ethical regulations.

## Abbreviations

AD: Alzheimer’s disease
CV: Coefficient of variation
FDR: False discovery rate
GLMM: Generalized linear mixed model
GSQS: Groningen sleep quality scale
LC: Locus coeruleus
MHPG: 3-Methoxy-4-hydroxyphenylethyleneglycol
MT-TFL: Magnetization transfer-weighted turbo flash
PT: Pontine tegmentum
p-tau_181_: Tau phosphorylated at threonine 181
*R*^2^_*β**_: Semi-partial *R*^*2*^
