# Supplementary material for: Associations between locus coeruleus integrity and nocturnal awakenings in the context of Alzheimer’s disease plasma biomarkers: a 7T MRI study
Source: Alzheimers Res Ther. 2021 Sep 24;13:159. doi: 10.1186/s13195-021-00902-8 (PMC8464124; doi:10.1186/s13195-021-00902-8)
Supplement: Supplementary file 1 — Additional file 1. Supplementary methods, figures, and tables. [file 13195_2021_902_MOESM1_ESM.docx]

**Additional File**

**Associations between locus coeruleus integrity and nocturnal awakenings in the context of Alzheimer’s disease plasma biomarkers: a 7T MRI study**

Maxime Van Egroo, PhD; Roy W.E. van Hooren, MSc; Heidi I.L. Jacobs, PhD

**Supplementary Methods.**

*Ultra-high field MRI acquisition procedure*

MRI data were acquired on a 7T Magnetom Siemens scanner (Siemens Healthineers, Erlangen, Germany). with a 32-channel head coil (Nova Medical, Wilmington, MA, USA). A whole-brain Magnetization Prepared 2 Rapid Acquisition Gradient Echoes (MP2RAGE^1^; TR = 5000 ms, TE = 2.47 ms, flip angle = 5°/3°, voxel size = 0.7 mm³, number of slices = 240) sequence was first acquired. Then, we performed an in-house developed magnetization transfer-weighted turbo flash (MT-TFL) sequence (TR = 538 ms, TE = 4.08, flip angle = 8°, voxel size = 0.4x0.4x0.5mm, number of slices = 60) with a field-of-view placed perpendicular to the pons and covering the area between the inferior colliculus and the inferior border of the pons. This sequence has been established to be particularly sensitive to LC-related contrast^2,3^, likely reflecting its neuronal and fiber projection density^4^.

*LC MRI processing pipeline*

Our 7T LC MRI processing pipeline involved the following steps: first, individual MT-TFL images were intensity-normalized by dividing by the subject-specific mean intensity of a 10x10 voxel region-of-interest in the pontine tegmentum (PT). For each participant, the PT region-of-interest was consistently placed in the axial slice which contained the highest intensity LC voxel. Importantly, no association was found between age and mean intensity in the PT, supporting that no age-related biases would be introduced by this normalization step. Second, a study-specific template was built from these individual intensity-normalized MT-TFL images using the *buildtemplateparallel* function from the Advanced Normalization Tools (ANTs)^5^ (transformation model and similarity metric used for registration = greedy SyN with cross-correlation). The LC was then manually delineated on the resulting template, based on voxel intensities and the anatomical properties of the LC. The LC mask was then applied on each individual intensity-normalized MT-TFL images registered to the study-specific template. Finally, median signal intensity across each slice along the vertical axis of the LC structure was extracted on a per hemisphere basis by applying the LC mask on individual intensity- and spatially-normalized images.

**Supplementary Table 1.** GLMM outputs of the associations between plasma biomarkers and subjective measures of sleep quality or nocturnal awakenings.

|  | ***Total tau*** | | ***p-Tau 181*** | |
| --- | --- | --- | --- | --- |
| Age | F_1,65_ = 0.37  *p* = 0.54 | F_1,65_ = 0.39  *p* = 0.53 | F_1,65_ = 8.28  *p* = 0.005  *R²_β*_* = 0.11 | F_1,65_ = 9.47  *p* = 0.003  *R²_β*_* = 0.13 |
| Sex | F_1,65_ = 1.02  *p* = 0.32 | F_1,65_ = 0.88  *p* = 0.35 | F_1,65_ = 2.17  *p* = 0.15 | F_1,65_ = 0.48  *p* = 0.49 |
| *APOE* status | F_1,65_ = 1.31  *p* = 0.26 | F_1,65_ = 1.23  *p* = 0.27 | F_1,65_ = 3.54  *p* = 0.07 | F_1,65_ = 4.14  *p* = 0.05 |
| Subjective sleep quality | F_1,65_ = 0.01  *p* = 0.93 |  | F_1,65_ = 1.81  *p* = 0.18 |  |
| Nocturnal awakenings |  | F_1,65_ = 0.02  *p* = 0.89 |  | F_1,65_ = 1.56  *p* = 0.22 |
|  | | | | |
|  | ***Aβ40*** | | ***Aβ42*** | |
| Age | F_1,65_ = 12.44  *p* < 0.001  *R²_β*_* = 0.16 | F_1,65_ = 11.45  *p* = 0.001  *R²_β*_* = 0.15 | F_1,65_ = 0.01  *p* = 0.95 | F_1,65_ = 0.01  *p* = 0.98 |
| Sex | F_1,65_ = 2.64  *p* = 0.11 | F_1,65_ = 3.87  *p* = 0.05 | F_1,65_ = 1.14  *p* = 0.29 | F_1,65_ = 1.20  *p* = 0.28 |
| *APOE* status | F_1,65_ = 1.37  *p* = 0.25 | F_1,65_ = 1.41  *p* = 0.24 | F_1,65_ = 10.31  *p* = 0.002  *R²_β*_* = 0.14 | F_1,65_ = 10.65  *p* = 0.002  *R²_β*_* = 0.14 |
| Subjective sleep quality | F_1,65_ = 0.75  *p* = 0.39 |  | F_1,65_ = 0.19  *p* = 0.66 |  |
| Nocturnal awakenings |  | F_1,65_ = 0.09  *p* = 0.76 |  | F_1,65_ = 0.09  *p* = 0.76 |

|  | ***Model 1*** | ***Model 2*** | ***Model 3*** |
| --- | --- | --- | --- |
| Middle-to-caudal LC integrity | F_1,63_ = 0.65  *p_FDR_* = 0.63 | F_1,63_ = 0.69  *p_FDR_* = 0.63 | F_1,61_ = 0.23  *p_FDR_* = 0.63 |
| Age | F_1,63_ = 0.01  *p* = 0.098 | F_1,63_ = 0.01  *p* = 0.93 | F_1,61_ = 0.03  *p* = 0.85 |
| Sex | F_1,63_ = 5.12  *p* = 0.03  *R²_β*_* = 0.08 | F_1,63_ = 5.32  *p* = 0.02  *R²_β*_* = 0.08 | F_1,61_ = 4.89  *p* = 0.03  *R²_β*_* = 0.07 |
| Depression | F_1,63_ = 25.31 *p* < 0.0001  *R²_β*_* = 0.29 | F_1,63_ = 24.69  *p* < 0.0001  *R²_β*_* = 0.28 | F_1,61_ = 24.11  *p* < 0.0001  *R²_β*_* = 0.28 |
| *APOE* status | F_1,63_ = 0.01  *p* = 0.99 | F_1,63_ = 0.01  *p* = 0.99 | F_1,61_ = 0.02  *p* = 0.90 |
| Middle-to-rostral LC integrity | F_1,63_ = 0.09  *p_FDR_* = 0.90 | F_1,63_ = 0.01  *p_FDR_* = 0.90 | F_1,62_ = 0.06  *p_FDR_* = 0.90 |
| Age | F_1,63_ = 0.01  *p* = 0.98 | F_1,63_ = 0.01 *p* = 0.99 | F_1,62_ = 0.02  *p* = 0.89 |
| Sex | F_1,63_ = 4.39  *p* = 0.04  *R²_β*_* = 0.07 | F_1,63_ = 4.64  *p* = 0.04  *R²_β*_* = 0.07 | F_1,62_ = 5.05  *p* = 0.03  *R²_β*_* = 0.08 |
| Depression | F_1,63_ = 25.26  *p* < 0.0001  *R²_β*_* = 0.29 | F_1,63_ = 25.05   *p* < 0.0001  *R²_β*_* = 0.28 | F_1,62_ = 24.33  *p* < 0.0001  *R²_β*_* = 0.28 |
| *APOE* status | F_1,63_ = 0.01  *p* = 0.97 | F_1,63_ = 0.01  *p* = 0.97 | F_1,62_ = 0.01  *p* = 0.97 |

**Supplementary Table 2.** GLMM outputs of the associations between subjective sleep quality (GSQS scores) and middle-to-caudal (top) or middle-to-rostral (bottom) LC structural integrity, considered bilaterally (model 1), for left LC (model 2), and for right LC (model 3).

**Supplementary Table 3.** GLMM outputs of the associations between subjective reports of nocturnal awakenings and the interaction term plasma biomarker*middle-to-caudal LC structural integrity, considered bilaterally.

|  | ***P-tau 181*** | ***Aβ40*** | ***Aβ42*** |
| --- | --- | --- | --- |
| Plasma marker*Middle-to-caudal LC integrity | F_1,61_ = 0.21 *p* = 0.65 | F_1,61_ = 0.02 *p* = 0.89 | F_1,61_ = 0.08 *p* = 0.78 |
| Middle-to-caudal LC integrity | F_1,61_ = 1.44 *p* = 0.23 | F_1,61_ = 0.16 *p* = 0.69 | F_1,61_ = 0.40 *p* = 0.53 |
| Plasma marker | F_1,61_ = 0.75 *p* = 0.39 | F_1,61_ = 0.01 *p* = 0.99 | F_1,61_ = 0.04 *p* = 0.85 |
| Age | F_1,61_ = 3.66 *p* = 0.06 | F_1,61_ = 1.39 *p* = 0.24 | F_1,61_ = 2.35 *p* = 0.13 |
| Sex | F_1,61_ = 6.83 *p* = 0.01  *R²_β*_* = 0.10 | F_1,61_ = 7.54 *p* = 0.008  *R²_β*_* = 0.11 | F_1,61_ = 7.41 *p* = 0.008  *R²_β*_* = 0.11 |
| Depression | F_1,61_ = 9.19 *p* = 0.004  *R²_β*_* = 0.13 | F_1,61_ = 8.13 *p* = 0.006  *R²_β*_* = 0.12 | F_1,61_ = 8.11 *p* = 0.006  *R²_β*_* = 0.12 |
| *APOE* status | F_1,61_ = 2.32 *p* = 0.13 | F_1,61_ = 1.50 *p* = 0.23 | F_1,61_ = 1.41 *p* = 0.24 |

**
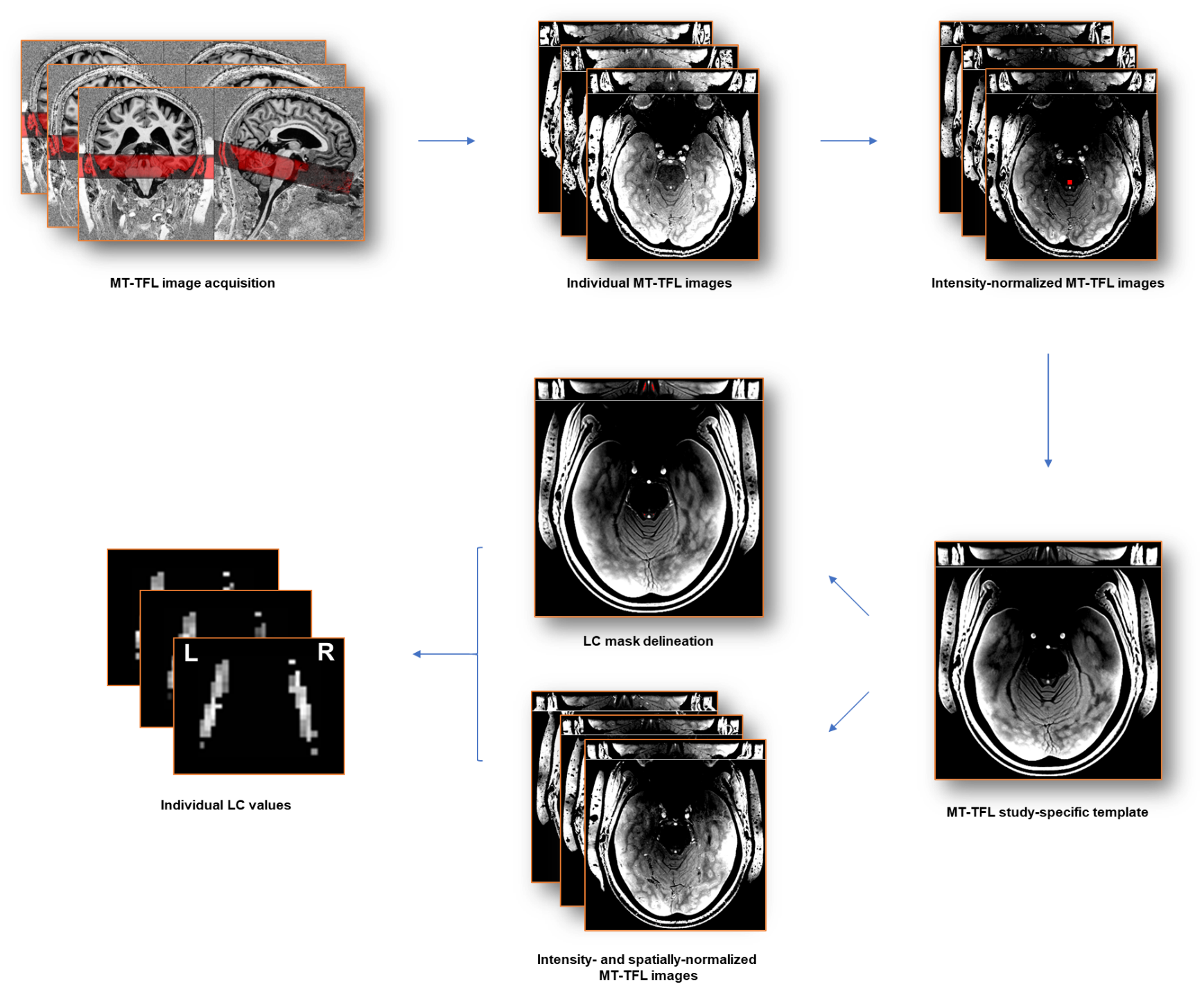
Supplementary Figure 1.** 7T LC MRI pre-processing pipeline. See **Supplementary Methods** for a full description of acquisition and pre-processing steps. The red square illustrates, for a representative participant, the 10x10 voxel region-of-interest located in the pontine tegmentum and used for subject-specific normalization of MRI signal intensity.

**Supplementary Figure 2.** *APOE* status vs. plasma biomarkers. * *p* < 0.05. After adjusting for age and sex, *APOE* ε4 carriers (29% of the sample) displayed significantly lower plasma levels of Aβ_42_ (F_1,66_=10.48, *p*=0.002, *R*²_β*_=0.14) and at-trend level higher plasma levels of p-tau_181_ (F_1,66_=3.44, *p*=0.07).

**
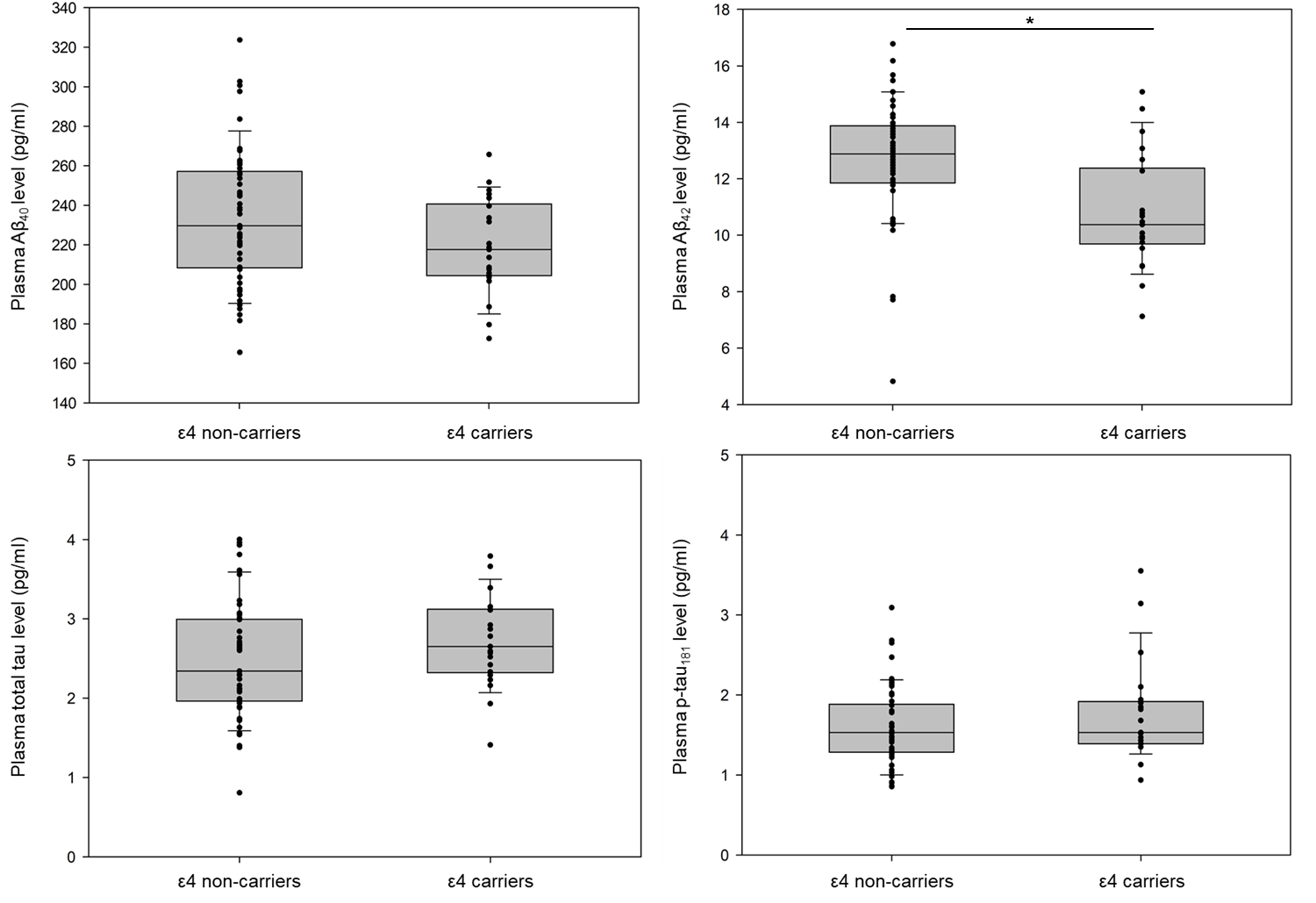
**

**Supplementary Figure 3.** Subjective sleep metrics vs. plasma biomarkers.

**
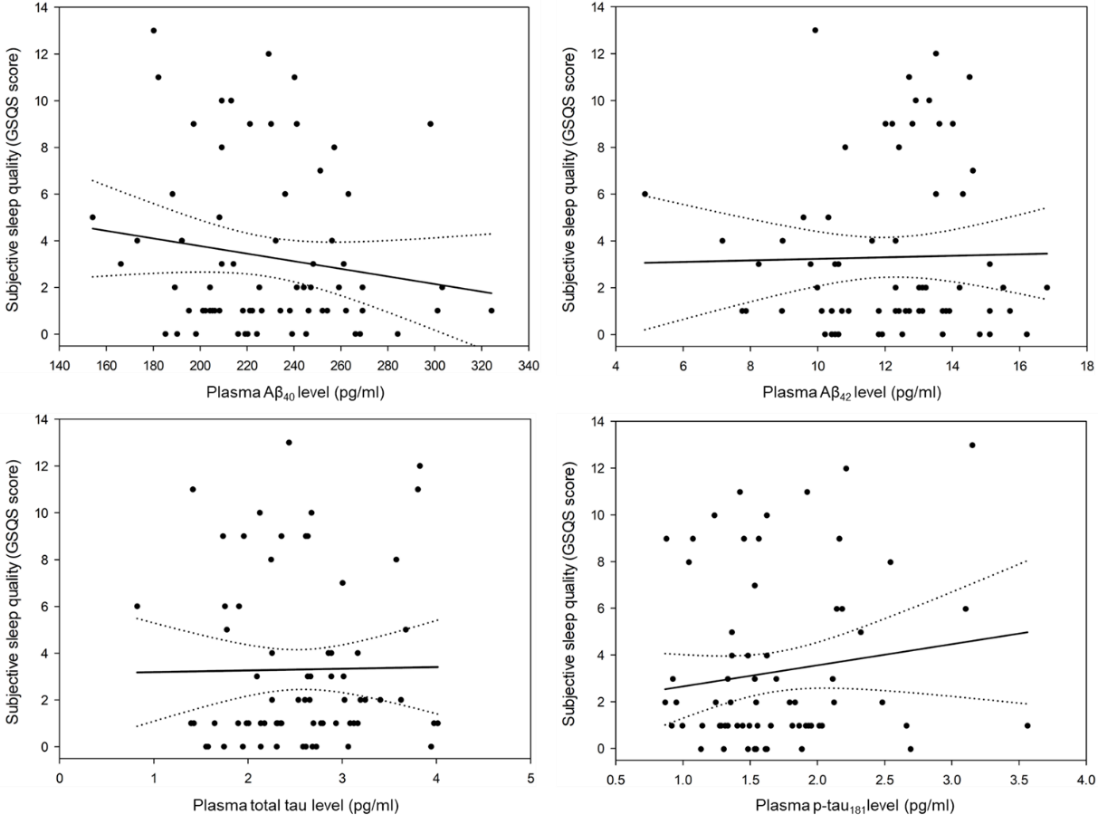
**Subjective sleep quality (GSQS score)

**
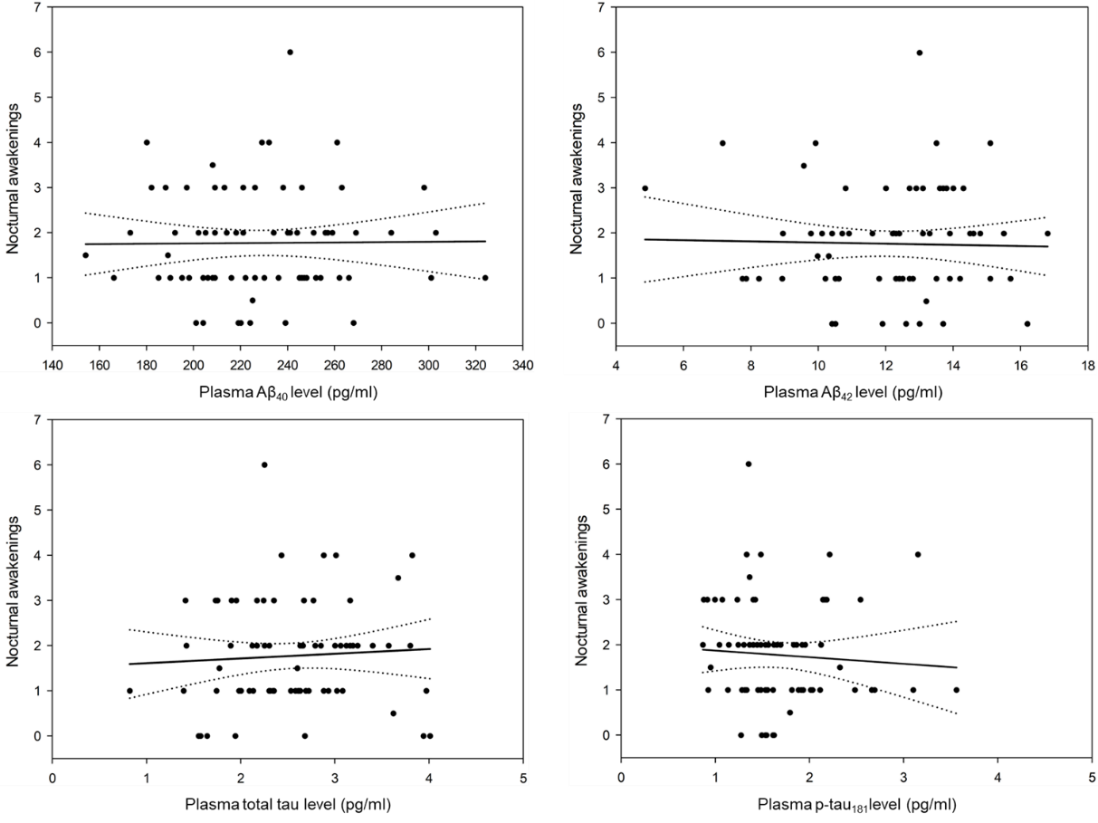
**Self-reported nocturnal awakenings

**Supplementary References**

1. Marques JP, Kober T, Krueger G, van der Zwaag W, Van de Moortele P-F, Gruetter R. MP2RAGE, a self bias-field corrected sequence for improved segmentation and T1-mapping at high field. *Neuroimage*. 2010;49(2):1271-1281. doi:10.1016/j.neuroimage.2009.10.002

2. Priovoulos N, Jacobs HIL, Ivanov D, Uludağ K, Verhey FRJ, Poser BA. High-resolution in vivo imaging of human locus coeruleus by magnetization transfer MRI at 3T and 7T. *Neuroimage*. 2018;168:427-436. doi:10.1016/j.neuroimage.2017.07.045

3. Jacobs HIL, Priovoulos N, Poser BA, et al. Dynamic behavior of the locus coeruleus during arousal-related memory processing in a multi-modal 7T fMRI paradigm. *Elife*. 2020;9:1-30. doi:10.7554/eLife.52059

4. Priovoulos N, van Boxel SCJ, Jacobs HIL, et al. Unraveling the contributions to the neuromelanin-MRI contrast. *Brain Struct Funct*. 2020;1(0123456789):3. doi:10.1007/s00429-020-02153-z

5. Avants BB, Yushkevich P, Pluta J, et al. The optimal template effect in hippocampus studies of diseased populations. *Neuroimage*. 2010;49(3):2457-2466. doi:10.1016/j.neuroimage.2009.09.062
